# Detect-S: an mHealth application to assist health professionals to identify suicide risk in hospitalized patients

**DOI:** 10.47626/2237-6089-2020-0079

**Published:** 2021-10-22

**Authors:** Ezequiel Teixeira Andreotti, Jaqueline Ramires Ipuchima, Marcos Vinicius Ludwig Pivetta, Angel Gabriel Arieta, Silvio César Cazella, Juliana Silva Herbert, Nicolas de Oliveira Cardoso, Wagner de Lara Machado, Ygor Arzeno Ferrão

**Affiliations:** 1 Universidade Federal de Ciências da Saúde de Porto Alegre Porto Alegre RS Brazil Universidade Federal de Ciências da Saúde de Porto Alegre (UFCSPA), Porto Alegre, RS, Brazil.; 2 Pontifícia Universidade Católica do Rio Grande do Sul Porto Alegre RS Brazil Pontifícia Universidade Católica do Rio Grande do Sul (PUCRS), Porto Alegre, RS, Brazil.

**Keywords:** Scale, suicide, assessment, questionnaire

## Abstract

**Introduction:**

Suicide is a serious public health problem that affects the whole world. This study describes development of the prototype for an mHealth application (app) intended to assist healthcare professionals to identify suicide risk in hospitalized patients and reports on testing of the app by some of these professionals, conducted to confirm its functionality.

**Method:**

This is applied exploratory research into use of Information Technology within the healthcare field, based on application prototyping for mobile devices. The research was conducted at the Universidade Federal de Ciências da Saúde de Porto Alegre (UFCSPA) from 2017 to 2019. Six healthcare professionals, one data scientist, and three undergraduate students in Biomedical Informatics took part in the study. All research participants signed the free and informed consent form.

**Results:**

The main findings show that the development team created a prototype named Detect-S, which became a cross-platform application (iOS and Android) offering 16 functions.

**Conclusion:**

It can be concluded that Detect-S has the potential to be a positive technological instrument that can be tested in a hospital setting to assist healthcare professionals to identify and manage patients with at risk of suicide.

## Introduction

Suicide is understood as intentional and conscious self-inflicted actions intended to result in death.^1^ In turn, suicidality refers to a wider concept that comprises various aspects of suicidal behavior, such as thinking of dying or killing oneself, suicidal plans, attempts, and completion of suicide.^2^

Approximately 800 thousand people die each year all over the globe due to this phenomenon, which represents one death every 35 seconds.^3^ In 2012, suicide accounted for 1.4% of all deaths in the world, being the 15th most common cause of death among the general population and the 2nd most common among people between 15 and 29 years old.^4^

The complexity of suicidality and its multiple causative factors may result in suicide being attempted in different environments, both private and public, and even inside hospitals.^5^ When suicide happens in a hospital setting, it does not solely impact on the victim and their family but also, and inevitably, on the healthcare professionals routinely active in that individual’s care – since these professionals are prone to facing such situations in the course of their jobs.^6^

Suicide episodes among hospitalized patients may also lead healthcare professionals to develop burnout syndrome and even depression, which cause higher rates of both absenteeism and presenteeism.^7^ However, even within a relatively controlled environment – as it is intended that hospitals should be – suicide is still difficult to prevent. Therefore, earlier identification of suicide ideation among hospitalized patients is also a key factor for preserving healthcare professionals’ mental health, while enabling effective preventative interventions targeting suicidal attempts and their outcomes.

Technological advances can contribute to clinical assessment of patients at risk of suicide. These technological aspects may contribute to improving the care provided by healthcare professionals to their patients. A variety of software and applications are being used to help with diagnosis of diseases and to improve people’s health. In this context, an application to assist with the early identification of suicide risk among hospitalized patients was developed.

Software is classified as an ‘intangible’ product, in which the main input is knowledge applied by specialized professionals and in which creativity and intellectual capacity allow development of adequate solutions for specific objectives. Its main feature is its flexibility, which has been incorporated into applications adapted to the needs of information management.^8^

Use of new technologies in the health field (e.g. mobile platforms) may contribute to making access to information simpler and more dynamic,^9^ allowing its applicability in a variety of settings and situations. The term “mobile-health” or “mHealth” was created by the World Health Organization and refers to use of mobile devices such as cell phones, patient monitoring devices, PDAs, and wireless devices in medical settings and in Public Health.^1^

When concepts like suicidality, working environment (e.g. hospital wards), and mobile technology are considered together, there seems to be a good opportunity for an application intended to assist health professionals to identify suicide risk at early stages, allowing development of suicide prevention strategies. Therefore, this study describes development of the prototype of a new mHealth application designed to assist healthcare professionals in identifying early suicide risk among hospitalized patients. Following the descriptions, healthcare professionals were asked to test the application in order to verify its functionality.

## Methodology

### Type of study

The study design is applied exploratory research investigating use of Information Technology in healthcare. The method employed was application prototyping for mobile devices. The systems development life cycle was used to develop the mobile app, which means that a series of phases were used, such as: requirement definition, analysis, design, development, testing, and prototyping.^10^

A new score scale for suicide risk assessment was created in order to be incorporated into the software. It is intended that the score will be tested *in loco* using the app, in a process that will be described in a future article. In this paper, besides the prototyping process, development of the new score scale will also be briefly described.

### Setting

The prototype was developed and tested at Universidade Federal de Ciências da Saúde de Porto Alegre (UFCSPA), between 2017 and 2019.

### Participants

The following participants took part in this research:

1. Software development: 1 psychiatrist, 1 registered nurse, 1 mental health nurse, 1 data scientist, and 3 Biomedical Informatics undergraduate students;
2. Healthcare professionals who participated in the software trial to test its functionality: 1 registered nurse, 1 mental health nurse, and 4 psychiatry residents.

### Ethical procedures

This project was approved by the UFCSPA Ethics Committee under registration number 2.387.612, in accordance with Brazilian National Health Council Resolution number 466/2012. All research participants signed free and informed consent forms.

### Suicidality Detection Score Scale (Detect-S)

First, we conducted a systematic literature review,^11^ based on Prisma principles,^12^ with the objective of identifying the most cited instruments for suicide risk assessment in the literature.

Following review of the articles identified, two (out of 20) instruments for assessing suicide risk were chosen because they seemed to be the ones most used: the Beck Scale for Suicide Ideation (BSI),^13^ which appeared 13 times among the 206 articles reviewed, and the Columbia-Suicide Severity Rating Scale (C-SSRS),^13^ which appeared 9 times among the 206 articles reviewed.^14^

The scales chosen (the BSI comprises 19 items and the C-SSRS 18 items) were analyzed by three healthcare professionals (1 Psychiatrist, 1 Registered Nurse, and 1 Mental Health Nurse). Following the first analysis, a new scale to detect suicide risk was developed – the Detect-S.

In addition to the questions from the existing scales, additional relevant clinical aspects were incorporated in the Detect-S scale, according to the authors’ clinical experience. This new instrument thus comprised 24 questions that cover suicidal intention, behavior, plan, and ideation, and was divided into three sections. The final score is calculated from the sum of each of the section scores.

The initial compilation of the Detect-S scale was sent using a Google Docs form to be analyzed and evaluated by experts with scientific production about suicide. These experts were selected based on their publishing about the subject over the last 10 years (from 2008 to 2018).

**Figure 1:**
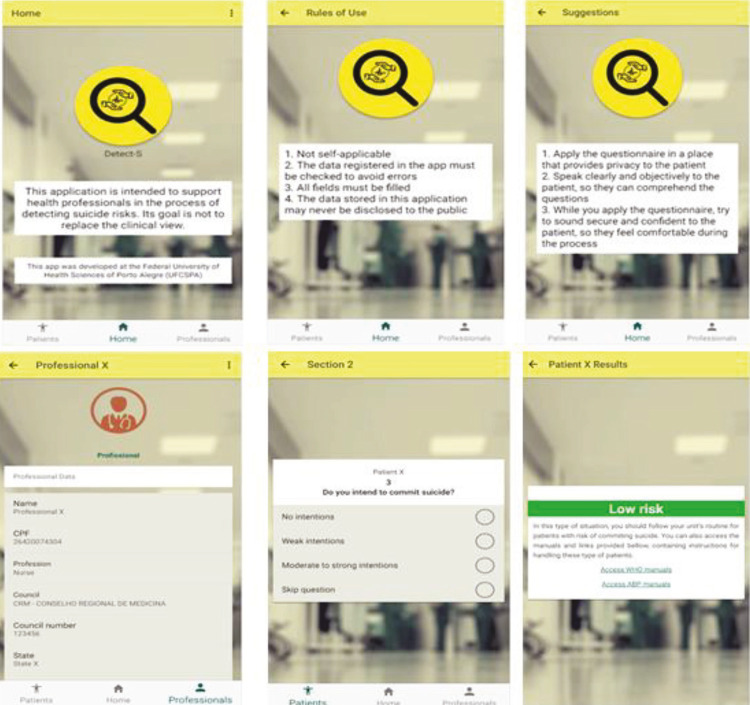
App interfaces.

The experts’ analysis yielded a few remarks related to the structure of the questions, which were intended to improve understanding both for the patient and the assessor. Some of these suggestions were incorporated to the Detect-S scale, resulting in the first version (Figure 1). Although the Beck Scale for Suicide Ideation (BSI) and the Columbia-Suicide Severity Rating Scale (C-SSRS) were created in other settings, they served as a basis for the researchers to create the Detect-S scale, which is a more complete instrument and will be tested in the hospital environment by health professionals using mobile devices. That application will be described in a future article, with the objective of verifying whether Detect-S constitutes an instrument that is applicable in hospital settings and comparing it with instruments already used in hospitals to assess suicide. The Detect-S scale questionnaire was adapted to be used as an application for mobile devices.

The software resulting from the Detect-S scale was installed on the healthcare professionals’/researchers’ mobile devices (cell phones or tablets) to evaluate its functionality, in order to obtain views and opinions that would be different from the developers’.

In the app, after suicidality and suicide risk have been assessed using the Detect-S scale, alerts are displayed on the screen indicating the priority for intervention for the patient assessed. Intervention priorities are coded using the same colors used in the Manchester Triage System (MTS)^15^: no risk – blue, low risk – green, moderate risk – yellow, and high risk – red.

### Development of the Detect-S mHealth Application

The stages of development of the prototype will be presented below.

### Definition of requirements (Functional and Non-Functional) and analysis

The functional requirements (RF) incorporated into the application are listed in Table 1.

**Table 1:** Functional requirements used in development of the Detect-S app

Code	Description	Priority
RF01	Add new patient to the system	Essential
RF02	Edit patient information in the system	Essential
RF03	Remove patient from the system	Essential
RF04	List the patients registered in the system	Essential
RF05	Export patient data to PDF and/or JSON formats	Essential
RF06	Add new professional	Essential
RF07	Edit professional information	Essential
RF08	Remove professional from the system	Essential
RF09	List professionals registered on the system	Essential
RF10	Export professional data to PDF and/or JSON formats	Essential
RF11	Show information from a specific patient: - Personal data; - Hospital setting.	Essential
RF12	Assess specific patient using the questionnaire: - Link professional to the application; - Tick each question choice; - Show questionnaire result.	Essential
RF13	Export data from a specific patient to PDF and/or JSON format.	Essential
RF14	Show information on a specific professional: - Personal data.	Essential
RF15	Export data from a specific professional to PDF and/or JSON format.	Essential
RF16	Show terms of use and recommendations.	Essential

RF = functional requirements.

A few non-functional requirements (RNF) are also listed in Table 2.

**Table 2:** Non-functional requirements used in development of the Detect-S application

Code	Description	Category
RNF01	Safely store data on the device to be analyzed later.	Reliability
RNF02	The software interface must have graphical elements displayed with wide dimensions and clear symbols, to minimize the risk of mistakes and misunderstanding.	Usability
RNF03	The system will be accessible through an offline application.	Availability
RNF04	The system will respond appropriately. Its interface must be adaptable to different interface sizes.	Portability
RNF05	The prototype will have satisfactory performance to reduce awkward situations for users.	Performance

RNF = non-functional requirements.

### Application development

The Ionic Cordova programming framework was used to develop the project. This program enables quick development, creation of a clear and functional design, and use of cross-platforms apps based on a single source code.^16^ A programming framework is an abstraction that supplies a template with several functions that can be used by a software developer.^17^ The framework in question does not have a steep learning curve, which reduces the time taken to generate a functional prototype.

### Outcomes

Some interfaces linked to provision of the functional requirements presented in Table 1 are described in this section, corresponding to the prototype developed.

### Welcome interface

The ‘home’ interface contains the following elements: a) the aim and the developers of the application, b) rules of use, and c) suggestions about how to use the questionnaire. The main objective of this first section is to prevent misuse of the app and remind the professional using it about best practices with regard to the patient, which incorporates functional requirement RF16.

### Patients interface

The ‘patients list’ interface lists all individuals who have been added and either have already answered or will answer the questionnaire. The professional using the app can expand patient information for each name by clicking on the ‘access’ option. This screen also provides the ‘add new patient’ (+ symbol) option, which enables the professional to access the ‘register new patient’ interface. This interface covers functional requirements RF1 and RF4.

### Professional interface

The ‘professional’ interface shows details of each professional registered on the system. The name of the professional whose information is being accessed is shown on the upper part of the interface. This encompasses functional requirement RF14.

### Questionnaire interface

The ‘questionnaire’ interface is made available after a professional has been linked to the application. This interface presents the full questionnaire and the response options. It encompasses functional requirement RF12.

The interface ‘results’ is made available after the questionnaire has been fully answered and displays the suicide risk score for the patient assessed, emphasized by the color established according to the Manchester Triage System. Suggestions for the healthcare professional on how to manage the patient are also shown. This interface encompasses functional requirement RF12. Figure 1 shows all of the application interfaces.

### Prototype trial by the developers

The prototype developers were: 1 Psychiatrist, 1 Registered Nurse, 1 Mental Health Nurse, 1 Data Scientist, and 3 undergraduate Biomedical Informatics students.

Before the end of development, the prototype was put through a phase of exploratory tests. In this phase, functionality tests were conducted in which testers used the app without being given instructions in advance, in order to test how they would respond to the app spontaneously. The functional elements in each interface (register fields, key buttons etc.) were tested in different ways, provoking software failure behavior (bugs) that was subsequently addressed.

The next test phase, described in the section below, considers the prospective app users.

### Prototype trial by healthcare professionals

Following the trial conducted by the developers, six healthcare professionals were invited to test the prototype (1 Registered Nurse, 1 Mental Health Nurse, and 4 Psychiatry residents). These professionals work at hospitals in which the application is intended to be used. The prototype was tested on the professionals’ cell phones and afterwards a structured questionnaire was administered at UFCSPA (in March 2019) in order to evaluate these professionals’ opinions of the software’s functionality. The results are shown in Table 3.

**Table 3:** Opinions of healthcare professionals who tested the Detect-S app

Assessors	Use	Design	Questions	Sensitivity	Specificity	Reliability
A	Excellent	Excellent	Excellent	Excellent	Good	Excellent
B	Excellent	Excellent	Excellent	Excellent	Excellent	Excellent
C	Excellent	Very good	Very good	Excellent	Excellent	Very good
D	Good	Good	Good	Good	Good	Very good
E	Very good	Excellent	Very good	Very good	Very good	Very good
F	Excellent	Very good	Excellent	Excellent	Very good	Excellent

Table 3 showed that 4 out of 6 (67%) healthcare professionals who used the Detect-S app considered its use was excellent; 3 out of 6 (50%) thought the design was excellent; 3 out of 6 (50%) said the questions were excellent; 4 out of 6 (67%) classified its sensitivity as excellent; 2 out of 6 (33%) rated its specificity as excellent, and 3 out of 6 (50%) rated the product’s reliability as excellent.

One of the questions on the structured questionnaire asked the testers their opinion about the product and any suggestions for improvement (‘what is your opinion about the product and what do you think could be done to improve it?’). Some of the answers were as follows: adjustments to questions about suicidal thoughts aiming to improve patient interpretation; display a message warning that the application does not replace an assessment by a qualified professional, but is rather intended to assist in early detection of suicide risk; fix bugs related to adding new patients to the app; and adjustment of interface transitions in order to maintain patients privacy. All of these suggestions were implemented to enhance the prototype.

MHealth technologies are constantly present in the daily lives of healthcare professionals.^18^ Therefore, creation and development of this prototype tends to add value to this healthcare context.

## Discussion

Based on the results obtained in this study, the Detect-S app is considered to be a promising tool to enhance the healthcare professionals’ practice, especially in hospital settings.

There are also other apps: Samaritans Radar, developed by the charity organization Samaritans for Twitter users, warns if an individual who follows a profile on the site intends to commit suicide. The Samaritans Radar software uses an algorithm for detecting keywords and phrases that signal this state, such as “tired of being alone,” “I hate myself,” “depressed,” “help me,” and “I need to talk to someone.” Users who have signed up to participate in the initiative will receive an email alert when someone makes this type of statement.^19^

Another application used is ADDS - Support for the Diagnosis of Depression and Assessment of Suicide Risk - Telessaúde/RS, designed with the objective of helping to diagnose depression and to define suicide risk, but which clearly states that it is not a replacement for clinical judgment. It is only compatible with IOS 6.0 or higher, on iPhone, iPad, or iPod Touch.^20^

However, both of the aforementioned applications are based only on risk of suicide due to depression and quantify the risk of a suicide attempt. They also limit availability to users, whether by requiring a Twitter account or because a current device is needed for the software to run. Finally, they also omit several variables from the risk assessment, such as: previous attempts to commit suicide, use of licit and/or illicit drugs, age, etc., and should not only focus on depression as the cause of the event, since it should be stressed that suicide is a multifactorial phenomenon.

Continuing this line of thought, the Detect-S application is an innovative product compared to the other applications mentioned, since it is a new tool capable of broadly and completely evaluating all psychopathological aspects of suicide, including the desire to die, suicidal ideation, attempted suicide, and severity and intensity of suicidal ideation and behavior. Detect-S makes a careful evaluation because it is based on a scale with 24 questions about suicide, whereas the other applications have six questions about the theme, which makes it difficult to make an effective assessment of the patient’s suicide risk. Moreover, only trained health professionals will administer Detect-S, whereas anyone can use the other software applications mentioned, which could generate the possibility of errors in suicide risk assessment.

The developers and assessors are in agreement that this prototype is extremely relevant for the healthcare field and for society in general. Development of this prototype resulted in a cross-platform application (iOS and Android) that encompasses 16 functions and can be used in hospital settings. During the trial phase, a number of aspects of the software (Detect-S) were identified as satisfactory, such as how easy it is to use the app, its design, the quality of questions, and its reliability, etc. During evaluation of its functions by healthcare professionals, several benefits of using this application were also praised, in terms of the idea and initiative to create such a prototype, as also mentioned above in the section ‘Prototype Trial by Healthcare Professionals’.

Use of mHealth solutions in hospital settings can result in simplified and more dynamic access to patients’ data, contributing to speeding up preventative actions to help the individual involved.^9^

### Limitations

This study and development of the prototype received no support from funding bodies, which was perceived as a limitation. The application was developed and is being improved by undergraduate students and health professionals who work in parallel with their agendas, which increased the time taken to complete the final version of the prototype.

Another important limitation of the study is that only six health professionals participated in the study, which made it difficult to apply the prototype, because it was difficult to find qualified professionals with time available to participate in the research, resulting in a need for additional time for software testing.

With regard to the three undergraduate Bioinformatics students who participated in development of the prototype, additional time was also needed to execute this stage, due to the difficulties finding dedicated and qualified students with time available for research.

### Future prospects

It is intended that the Detect-S scale will be validated and the Detect-S app tested in a hospital setting in a future article, in which the app will be used to assess risk of suicide with the scale. This is expected to assist healthcare professionals to customize care for the patients according to messages and suggestions displayed by the software after scores have been calculated.

It is hoped that this study will inspire further research and creation of other products to help healthcare professionals with their practice in relation to suicide and suicidality, emphasizing the importance of mHealth technologies in this context.

## Conclusion

This application can be considered an innovative and technological solution to help healthcare professionals detect suicide risk among hospitalized patients early.

This mHealth prototype was sent to the Innovations Centre of the Universidade Federal de Ciências da Saúde de Porto Alegre – UFCSPA (NITE-Saúde) seeking to guarantee its patent. The patent request was registered with the Brazilian National Institute for Industrial Property (INPI) in March 2019.

As a future prospect, now that the application is complete, administration of Detect-S in hospitalized patients will be addressed in another article.
